# Using CRISPR-Cas9 Technology to Eliminate Xyloglucan in Tobacco Cell Walls and Change the Uptake and Translocation of Inorganic Arsenic

**DOI:** 10.3389/fpls.2022.827453

**Published:** 2022-02-16

**Authors:** Meng Wang, Xinxin Song, Shuaiqiang Guo, Peiyao Li, Zongchang Xu, Hua Xu, Anming Ding, Rana Imtiaz Ahmed, Gongke Zhou, Malcom O’Neill, Dahai Yang, Yingzhen Kong

**Affiliations:** ^1^College of Agronomy, Qingdao Agricultural University, Qingdao, China; ^2^Key Laboratory of Tobacco Gene Resources, Tobacco Research Institute, Chinese Academy of Agricultural Sciences, Qingdao, China; ^3^Key Laboratory of Biofuels, Qingdao Engineering Research Center of Biomass Resources and Environment, Shandong Provincial Key Laboratory of Energy Genetics, Qingdao Institute of Bioenergy and Bioprocess Technology, Chinese Academy of Sciences, Qingdao, China; ^4^College of Resources and Environment, Qingdao Agricultural University, Qingdao, China; ^5^Academy of Dongying Efficient Agricultural Technology and Industry on Saline and Alkaline Land in Collaboration With Qingdao Agricultural University, Dongying, China; ^6^Complex Carbohydrate Research Center, University of Georgia, Athens, GA, United States; ^7^China Tobacco Breeding and Biotechnology Research Center, Yunnan Academy of Tobacco Agricultural Sciences, Kunming, China

**Keywords:** cell wall, xyloglucan xylosyltransferases, CRISPR-Cas9, arsenic, uptake

## Abstract

Xyloglucan is a quantitatively major polysaccharide in the primary cell walls of flowering plants and has been reported to affect plants’ ability to tolerate toxic elements. However, it is not known if altering the amounts of xyloglucan in the wall influences the uptake and translocation of inorganic arsenic (As). Here, we identified two *Nicotiana tabacum* genes that encode xyloglucan-specific xylosyltransferases (XXT), which we named NtXXT1 and NtXXT2. We used CRISPR-Cas9 technology to generate *ntxxt1*, *ntxxt2*, and *ntxxt1/2* mutant tobacco plants to determine if preventing xyloglucan synthesis affects plant growth and their ability to accumulate As. We show that NtXXT1 and NtXXT2 are required for xyloglucan biosynthesis because no discernible amounts of xyloglucan were present in the cell walls of the *ntxxt1/2* double mutant. The tobacco double mutant (*ntxxt1/2*) and the corresponding Arabidopsis mutant (*atxxt1/2*) do not have severe growth defects but do have a short root hair phenotype and a slow growth rate. This phenotype is rescued by overexpressing *NtXXT1* or *NtXXT2* in *atxxt1/2*. Growing *ntxxt* mutants in the presence of AsIII or AsV showed that the absence of cell wall xyloglucan affects the accumulation and translocation of As. Most notably, root retention of As increased substantially and the amounts of As translocated to the shoots decreased in *ntxxt1/2*. Our results suggest that xyloglucan-deficient plants provide a strategy for the phytoremediation of As contaminated soils.

## Introduction

The polysaccharide-rich cell wall is plants’ first line of defense against toxic elements present in soils and water as it may prevent them from entering the cytoplasm (Parrotta et al., 2015). For example, flax hypocotyls adapt to the “b” class soft metal cadmium (Cd^2+^) by changing the methyl-esterification pattern of homogalacturonan (Douchiche et al., 2010). Increases in pectin and hemicellulose in rice leaves have been reported to be responsible for increased Cd^2+^ accumulation in root cell walls and a decrease in soluble Cd^2+^ (Xiong et al., 2009). However, a decrease of pectin and hemicelluloses resulting from phosphorous deficiency has also been reported to enhance Cd^2+^ exclusion in Arabidopsis root walls (Zhu et al., 2012a). Cd^2+^ tolerance in tobacco has been increased by overexpressing a *Populus euphratica* gene encoding a xyloglucan (XyG) endotransglucosylase/hydrolase (XTH) to decrease the amount of wall XyG (Han et al., 2014). A decrease of XyG (*xth31* mutant; Han et al., 2014) or the absence of XyG (*xxt1/2* double mutant; Zhu et al., 2012b) reduces the accumulation of the “a” class hard metal aluminum (Al) in Arabidopsis root cell walls. The Al-binding capacity is determined in part by the extent of XyG *O*-acetylation, since more Al accumulates in the walls of Arabidopsis XyG *O*-acetyltransferase mutants than their wild-type counterpart (Zhu et al., 2014).

The primary cell wall is a versatile and dynamic structure (Fry, 1990). It provides mechanical strength yet is capable of expanding to allow cell growth and also has roles in water and mineral uptake, pathogen resistance, as well as developmental and physiological processes. Cell walls have become a target for bioengineering to improve the value of biomass for renewable energy production (Loque et al., 2015) or to enhance a plants’ ability to bind toxic element present in contaminated soils (Zhu et al., 2012a,b, 2014; Han et al., 2014).

XyG is present in the cell walls of all land plants and is one of the most abundant non-cellulosic polysaccharides in dicot primary walls (Pauly and Keegstra, 2016). Xyloglucans have a 1,4-linked β-d-glucan backbone with α-d-xylopyranosyl (Xyl*p*) residues attached at *O*-6. The xylosyl residue is often substituted with d- and l-galactosyl, l-fucosyl, d-galacturonosyl, l-arabinopyranosyl, and/or l-arabinofuranosyl moieties (Pauly and Keegstra, 2016). To date, 24 different side chains, which are described using a one letter code have been identified (Pauly and Keegstra, 2016). The type and order of XyG sidechains depend on the plant species, the tissue and cell type, and the developmental state of the cell (Pauly and Keegstra, 2016). XyGs are classified either as “XXXG type” or “XXGG type.” Tobacco leaf XyG is composed predominantly of XXGG and XSGG subunits. S represents an α-l-Ara*f*-(1 → 2)-α-d Xyl*p*-[1 → side chain linked to *O*-6 of a 4-linked β-d-Glc*p* residue (Hoffman et al., 2015)].

The biosynthesis of XyG has been studied in detail (Pauly and Keegstra, 2016). Xyloglucan-specific xylosyltransferases (XXTs) catalyze the addition of Xyl residues to *O*-6 of the glucan backbone (Cavalier and Keegstra, 2006; Zabotina, 2012; Culbertson et al., 2016). In Arabidopsis, the XXT genes belong to the GT34 family which containing AtXXT1 to AtXXT5 and two mannan:galactosyltransferases (MGs) AtGT6 and AtGT7 (Vuttipongchaikij et al., 2012). In Arabidopsis, XXT1 and XXT2 are responsible for the most, if not all, of this xylosylation (Zabotina et al., 2012), since no XyG is discernible in the walls of the *xxt1/2* double mutant (Cavalier et al., 2008). This mutant grows and develops normally, which has led plant scientists to question the notion that a cellulose-XyG network is the major load-bearing structure in the walls of growing plant cells (Schultink et al., 2014). Nevertheless, the *xxt1/2* double mutant does have some visible phenotypes including short root hairs with bulging bases (Cavalier et al., 2008), shorter and wider hypocotyls, and bent stems (Xiao et al., 2016). Such changes are consistent with the notion that some of the cells of the *xxt1/2* mutant have walls with altered mechanical and chemical properties.

Arsenic (As) is a naturally occurring toxic metalloid element ranked as a top 20 priority hazardous substance by the Agency for Toxic Substances and Disease Registry (Abbas et al., 2018). Most environmental As occurs as oxyanions in either of two oxidation states: +3 (AsIII, arsenites) or +5 (AsV, arsenates and organoarsenic compounds). Low amounts of AsIII or AsV may cause substantial morphological, physiological, and biochemical changes once they enter plant cells (18). These include stunted growth, reduced photosynthetic efficiency, and decreased biomass accumulation. Arsenic also causes increased generation of reactive oxygen species (ROS), which interferes with numerous metabolic pathways (Abbas et al., 2018). The As absorbed by plants may accumulate to toxic levels in vegetables, grains, and fruits (Chen et al., 2017a), which can lead to As poisoning in humans *via* the food chain.

It is not known if the uptake or accumulation of As by plants is affected by cell wall XyG or whether XyG can lessen the deleterious effect of this toxic element. Common tobacco (*Nicotiana tabacum*) is an important agricultural crop and a model plant widely used for studying fundamental biological processes (Sierro et al., 2014). The genome of *Nicotiana tabacum* was sequenced in 2014 (Sierro et al., 2014). In this study, we identified 11 *XXT* orthologs in the tobacco genome. Two of these genes, *NtXXT1* and *NtXXT2*, are required for xyloglucan biosynthesis. CRISPR-Cas9 technology was used to generate single (*ntxxt1* and *ntxxt2*) and double (*ntxxt1/2*) mutants. The double mutant lacked XyG in its cell walls. We show that eliminating XyG increases the amounts of As bound to the root walls, which results in less As being translocated to the tobacco shoots.

## Materials and Methods

### Plant Materials and Growth Conditions

*N. tabacum* var. K326 was used for genetic transformations. The seeds of K326 and the *xxt* mutants were surface sterilized with aq. 10% sodium hypochlorite (NaClO) and then with aq. 75% alcohol. The seeds were germinated on half-strength Murashige and Skoog (MS) medium. Two-week-old seedlings were transferred to potting soil and grown at 25 ± 1°C with a long-day photoperiod (16-h light/8-h dark) and 60% humidity in a greenhouse.

### NtXXT Identification and Phylogenetic Analysis

XXT orthologs in *N. tabacum* were identified using the protein sequences of AtXXT1 to AtXXT5 and AtGT6 to AtGT7 downloaded from TAIR^1^ and used as queries to BLAST the International Tomato Genome Database.^2^ *AtXXTs* and *NtXXTs* protein sequences were aligned with ClustalW2^3^ with a gap extension penalty of 0.1. Redundant sequences of NtXXTs were excluded. Phylogenetic analysis with a Poisson model method was performed using MEGA6.0 with a neighbor-joining method. The robustness of the tree topology was assessed using 1,000 bootstrap replicates.

### Tissue Expression Analysis of *NtXXTs*

The spatiotemporal expression patterns of *NtXXTs* were obtained using semi-quantitative PCR. Selected tissues (young leaves, mature leaves, and senescent leaves, stem, roots, vein, and flowers) were collected during the tobacco growing season. Three biological independent replicates of each tissue were used. The tissues were frozen in liquid nitrogen and kept at −80°C. RNA extraction, cDNA synthesis, and semi-quantitative PCR reactions were performed as described (Wang et al., 2018). Tobacco *NtACTIN1* (XM_019370655.1) was used as the reference gene (Wang et al., 2018).

### Single Guide RNA Design and Construction of CRISPR-Cas9 Binary Vectors

Single guide RNA (sgRNA) sequences of *Ntab-K326_AWOJ-SS412* (*NtXXT1*) and *Ntab-K326_AWOJ-SS848* (*NtXXT2*) were designed with the web-based tool CRISPR MultiTargeter.^4^ Three different sgRNA sequences were designed for each gene to improve the success rate of editing. Each sgRNA sequence contained 20 nucleotides followed by the NGG trinucleotide protospacer adjacent motif (PAM) at the 3′-end of the target region. A pair of complementary sgRNA DNA oligonucleotides were synthesized in Qingke sequencing company and annealed to generate dimers. The dimers were inserted into a modified CRISPR-Cas9 pORE vector plasmid driven by the Arabidopsis U6-26 promoter (Gao et al., 2015). PCR and Sanger sequencing were used to ensure that no polymorphisms existed between the sgRNAs and the corresponding target sequences.

### Tobacco Leaf Cell Transformation by *Agrobacterium* Infiltration

Tobacco leaf disc were used for transformation. *Agrobacterium tumefaciens* strain LBA4404 containing the CRISPR-Cas9 plasmid constructs was grown at 28°C in Luria–Bertani medium containing rifampicin and kanamycin antibiotics to an OD_600_ of 0.8. The bacteria were collected by centrifugation and the pellet suspended in MS liquid medium containing 30 g/L sucrose and 20 mg/L acetosyringone (MS_0_ medium) and adjusted to an OD_600_ of 0.8. Tobacco leaf discs were then immersed for 8 to 10 min in the MS_0_ medium containing the bacteria. The leaf discs were transferred to antibiotic-free MS medium and kept for 3 days in the absence of light. The transfected discs were then transferred to differentiation medium (MS containing 2 mg/L 6-benzylaminopurine, 0.1 mg/L 1-naphthaleneacetic acid [NAA], 50 mg/L kanamycin and hygromycin, and 500 mg/L cefotaxime) until buds formed. The regenerated plantlets were transferred on root-inducing medium (MS + 0.1 mg/L NAA + hygromycin + 200 mg/L cefotaxime) to produce the roots. Lastly, the drug-resistant seedlings were planting in the soil and used for further analysis.

### Monosaccharide Compositions of the Cell Walls

The leaves of 6-week-old tobacco plants (K326, *ntxxxt1*, *ntxxt2*, and *ntxxt1/2*) were collected, frozen in liquid nitrogen, and ground to a powder. The materials were then prepared as their alcohol-insoluble residues (AIR) as described (Pena et al., 2008). In brief, the powder was sequentially extracted for 30 min each with aqueous 70 and 80% ethanol and then with absolute ethanol. The residue was suspended in acetone, filtered through Whatmans filter paper, and allowed to dry in a fume hood. The AIR was de-starched with α-amylase and amyloglucosidase (Sigma–Aldrich, St. Louis, MO, United States) at 37°C overnight.

Three different AIR (2 mg) from each plants was hydrolyzed for 2 h at 120°C with 2 M trifluoroacetic acid (TFA). The hydrolysates were then reacted for 30 min at 70°C with 1-phenyl-3-methyl-5-pyrazolone (PMP). The mixture was extracted three times with chloroform and the PMP-monosaccharides were analyzed by high-performance liquid chromatography (HPLC; Wang et al., 2020). Three biological replicates were used for each sample.

### Matrix-Assisted Laser Desorption Ionization Time-of-Flight Mass Spectrometry

The de-starched AIRs prepared from K326, *ntxxt1*, *ntxxt2*, and *ntxxt1/2* leaves were treated with 50 mM ammonium oxalate to solubilize pectin and then with 4 M KOH to solubilize XyG. The XyG subunit composition (Kong et al., 2015) was determined by treating the 4 M KOH-soluble material in 50 mM ammonium formate, pH 5, for 24 h at room temperature with two units XEG (Kong et al., 2015). Ethanol was then added to 70% (v/v). The suspension was centrifuged (2000 × *g*) and the soluble fraction transferred to a clean tube and concentrated to dryness under a flow of warm air. To ensure the ammonium formate was completely removed the residue was dissolved in water and freeze-dried three times. Solutions of the XEG-treated material in water (~1 mg/ml) were analyzed using a Bruker Microflex LT matrix-assisted laser desorption ionization time-of-flight mass spectrometry (MALDI-TOF-MS) in the positive ion mode and Bruker workstation (Bruker, Billerica, MA, United States) as described (Kong et al., 2015).

### Arsenic Absorption by Tobacco Plants

Six-week-old tobacco were watering the HoaglandArnon nutrient solution (HNS) containing 20 μM arsenite (AsIII, NaAsO_2_) or 20 μM arsenate (AsV, Na_2_HAsO_4_·7H_2_O) and cultivated for 3 days. The roots, shoots, and leaves were separately collected and kept at −80°C. For determining the As concentration in roots and shoots, the frozen tissues (~10 g) were kept for 30 min at 120°C, and then at 75°C until they reached a constant weight. For determining the As concentration in roots cell walls and leaves cell walls, the frozen tissues were grounded into powders and extracted in subcellular extraction buffer (250 mmol/L sucrose, 50 mmol/L Tris-HCl (pH7.4), and 1 mmol/L erythritol dithiocarcinol). After centrifugation at 3,000 rpm for 15 min, the cell wall components were precipitated and then dried at 75°C. Approximately 0.5 g of dry materials (three replicates performed in each plants) were digested in concentrated nitric acid (4 ml) for overnight. H_2_O_2_ (2 ml) was added, and the mixture was kept for 4 h at 120–130°C. After being cooled, the solution were then heated at 180–230°C to heat acid. The remaining solution was used to determine the As concentration by inductively coupled plasma mass spectrometry (ICP-MS; United States, PerkinElmer; Chen et al., 2016). This experiment was repeated three times.

## Results

### Expression Profiles of Xyloglucan Xylosyltransferase Orthologs in Tobacco

**Figure 1:**
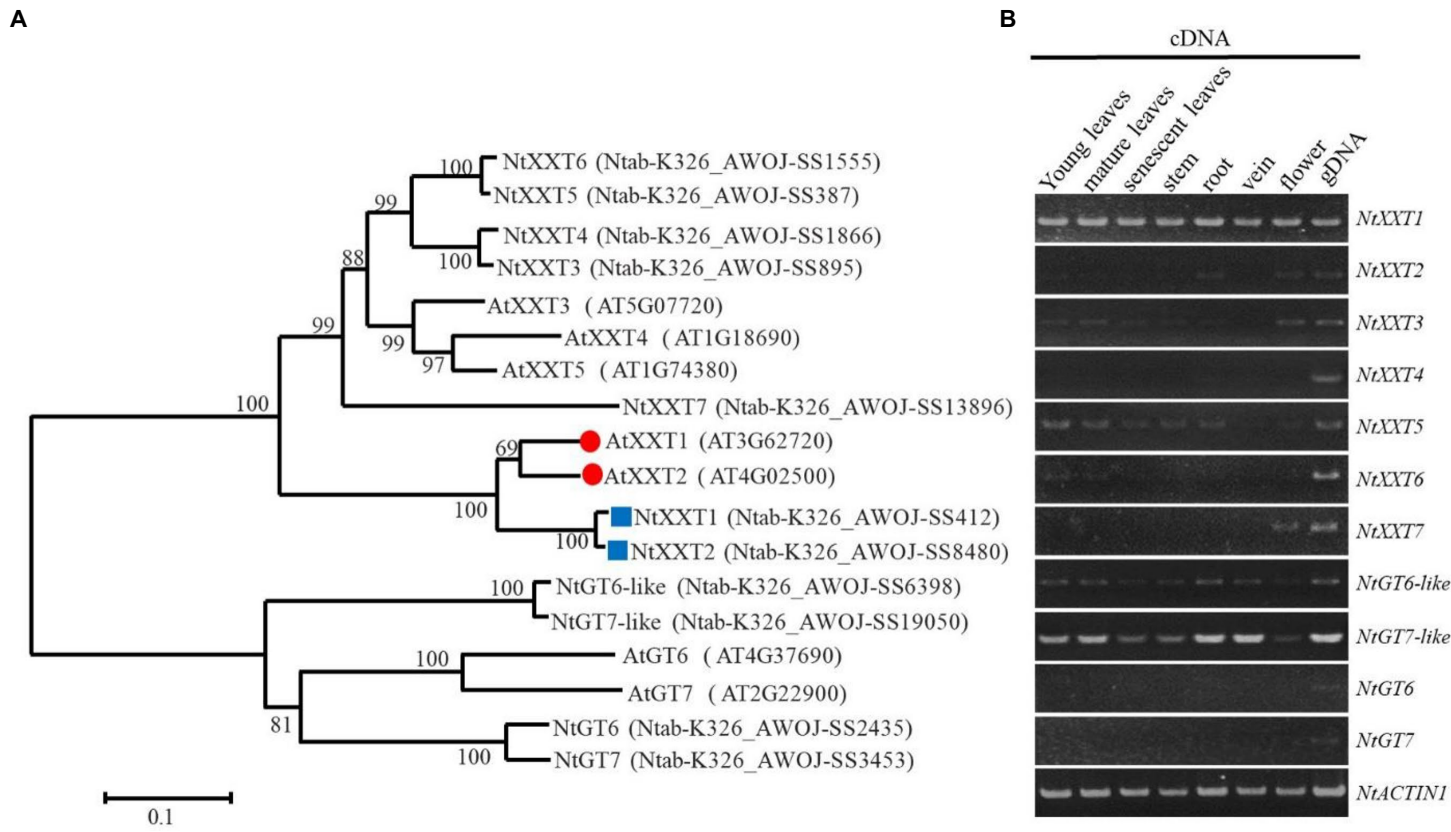
Phylogenetic analysis of NtXXT and AtXXT protein sequences and tissue-specific expression of the corresponding *NtXXT* genes. **(A)** Phylogenetic analysis of the AtXXT and NtXXT, NtGT protein sequences. The red circles indicated the genes that been well studied in *Arabidopsis*. The blue boxes indicated the NtXXTs that are tightly closed to the well-studied AtXXTs. **(B)** The expression pattern of all the *NtXXTs* in different stages of leaves, stems, roots, vein, and flowers.

A total of 11 putative *XXTs* genes were identified in the *N. tabacum* L. genome. Our phylogenetic analysis classified the *NtXXTs* into two sub-clusters. Seven *NtXXTs* (*NtXXT1* to *NtXXT7*) were clustered together with all five *AtXXTs*. Surprisingly, the remaining four *NtXXTs* (*NtXXT8* to *NtXXT11*) were clustered into a second group with MGs AtGT6 and AtGT7 (Figure 1A). Thus, these four genes are putative mannan:galactosyltransferases (MGs), and we renamed them as *NtGT6-like*, *NtGt7-like*, *NtGT6*, and *NtGT7*, respectively. *Ntab-K326_AWOJ-SS412* and *Ntab-K326_AWOJ-SS848* were grouped into the same branch as *AtXXT1* and *AtXXT2*, so we named them *NtXXT1* and *NtXXT2*, respectively, (Figure 1A).

We used semi-quantitative PCR to explore the expression levels of the *NtXXTs* in young leaves, mature leaves, senescent leaves, stem, roots, vein, and flowers. The expression patterns of *NtXXTs* varied somewhat. *NtXXT1* and *NtGT7-like* were expressed at a relatively high level in all tissues, whereas *NtXXT3*, *NtXXT5*, and *NtGT6-like* showed relatively low expression level in all tissues. The expression of *NtXXT4*, *NtGT6*, and *NtGT7* was barely detectable in any of the tissues (Figure 1B). NtXXT2 showed weak expression in leaves, stem, roots, and flowers (Figure 1B).

### CRISPR-Cas9 Induced Mutation of Tobacco XXTs

Based on our phylogenetic and expression pattern analyses, we hypothesized that NtXXT1 and NtXXT2 are required for xyloglucan biosynthesis. To test this, we generated *ntxxt1* and *ntxxt2* knockout mutants by transforming K326 plants with pORE vectors that contained the Cas9 gene and sequences for single-stranded guide RNAs (sgRNA) that target *NtXXT1* and *NtXXT2* individually.

**Figure 2:**
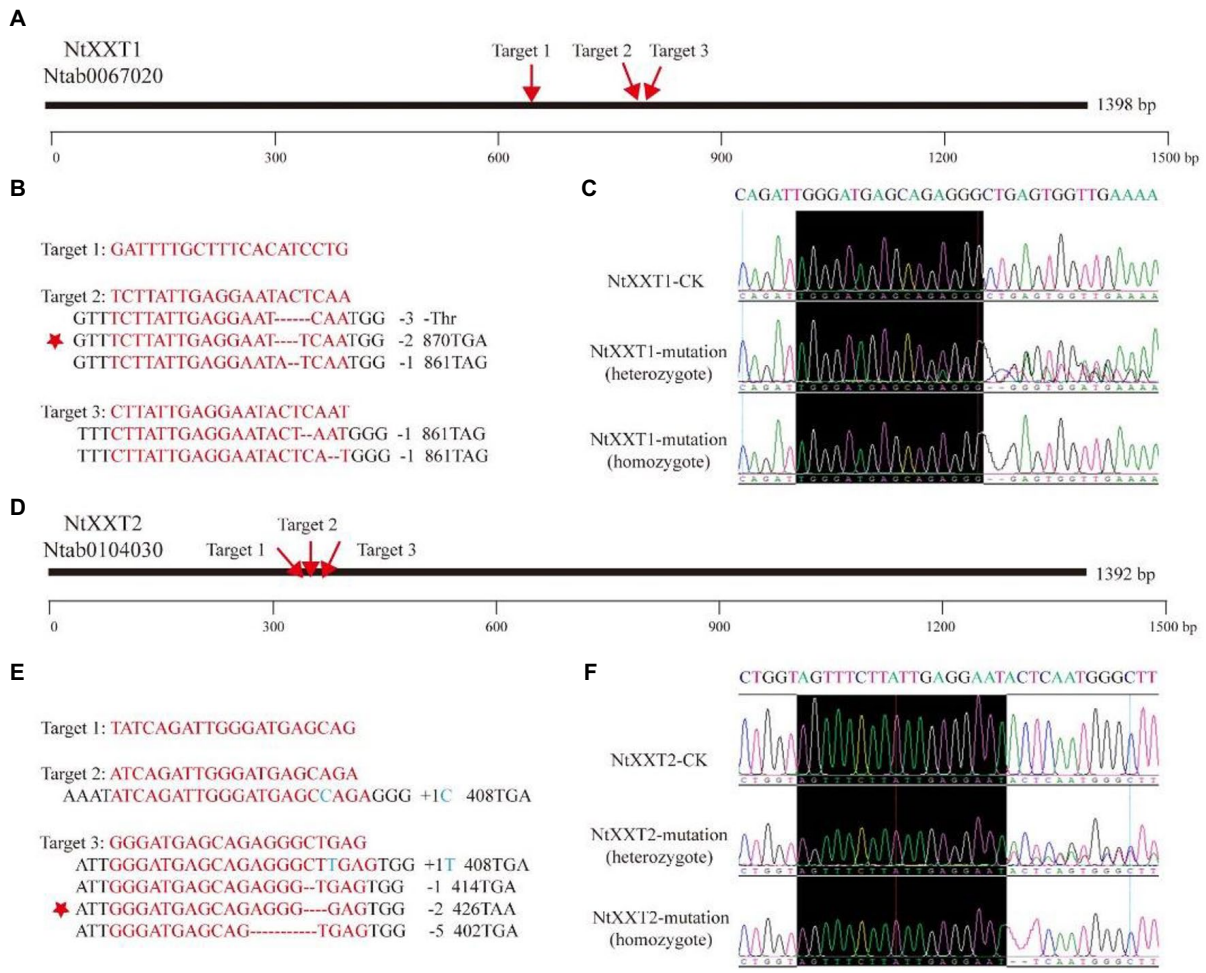
Regions of *NtXXT1* and *NtXXT2* targeted by CRISPR-Cas9. **(A)** The gRNA targets in the *NtXXT1*. The red arrows indicate the positions of the three target sites. **(B)** Editing profiles at *NtXXT1*. The target sequence is shown in red letters, for which red dashes indicated deletions. Different editing types were detected which can result in amino acid deletion and termination codon. The red stars indicated the deletion lines which were obtained homozygous lines and used for further analysis. **(C)** The knockout of *NtXXT1* was verified by gold-standard Sanger sequencing in heterozygous and homozygous lines which comes from the red star labeling in **(B)**. The black box indicated the target sequence upstream of the editing location. **(D)** The gRNA targets in the *NtXXT2*. The red arrows indicate the positions of the three target sites. **(E)** Editing profiles at *NtXXT2*. The target sequence is shown in red letters, for which red dashes indicated deletions, and the blue letters indicated insertion. Different editing types were detected which can result in termination codon. The red stars indicated the deletion lines which were obtained homozygous lines and used for further analysis. **(F)** The knockout of *NtXXT2* was verified by gold-standard Sanger sequencing in heterozygous and homozygous lines. The black box indicated the target sequence upstream of the editing location.

Three sgRNAs were designed for each gene (Figure 2). A total of 43 and 60 transgenic plants for targets 2 and 3, respectively, were obtained for *NtXXT1*. No transgenic plants were obtained for target 1. PCR sequencing revealed that four individuals had base variations at target 2 and two at target 3, with editing efficiencies of 9 and 3%, respectively. These transgenic plants showed deletion of one to three bases in the target region of *NtXXT1* (Figure 2B).

A total of 49, 19, and 82 transgenic plants were obtained for targets 1, 2, and 3, respectively, of *NtXXT2*. However, no plants with base changes at the target 1 site were identified. One target 2 transgenic plant and three target 3 transgenic plants were identified with editing efficiencies of 5 and 4%, respectively. Deletions of one to five bases and base insertions were identified at the target 2 and 3 regions of *NtXXT2* (Figure 2E). All the indel edit types resulted in termination codon occurrences in subsequent sequences both in *NtXXT1* and *NtXXT2* (Figures 2B,E). After self-fertilization, the F2 generation plants were used to screen for homozygous plants that lack Cas9. Only the “AC” (Adenine deoxyribonucleotide and Cytosine deoxyribonucleotide) and “CT” (Cytosine deoxyribonucleotide and Thymine deoxyribonucleotide) deletion homozygote plants of *NtXXT1* and *NtXXT2* (Figures 2C,F) were obtained and were referred to as knockout lines *ntxxt1* and *ntxxt2*, respectively. The *ntxxt1/2* double mutant was obtained by crossing the homozygous *ntxxt1* and *ntxxt2* mutants. The homozygous *ntxxt1/2* double mutant was identified by gold-standard Sanger sequencing.

### The *ntxxt1/2* Mutant Has Growth Slower Defects and Has a Severe Root Hair Phenotype

**Figure 3:**
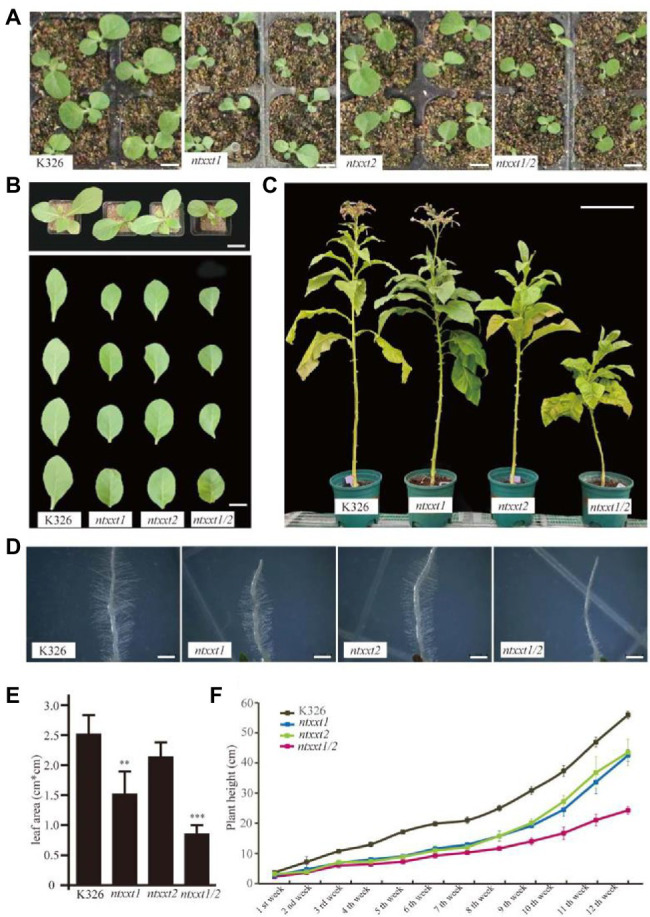
The morphology phenotype of *ntxx1/2* double mutants. **(A)** Two-week-old seedlings of K326, *ntxxt1*, *ntxxt2*, and *ntxxt1/2* mutants. Bar = 1.5 cm. **(B)** The morphological phenotypes and leaf shape of K326, *ntxxt1*, *ntxxt2*, and *ntxxt1/2* 4-week-old seedlings. Bar = 3.5 cm. **(C)** The morphological phenotypes of K326, *ntxxt1*, *ntxxt2*, and *ntxxt1/2* adult plants. Bar = 0.25 m. **(D)** Root hair phenotypes of K326, *ntxxt1*, *ntxxt2*, and *ntxxt1/2* seedlings. Bar = 0.5 mm. **(E)** The statistical data leaf area of the 2-week-old K326, *ntxxt1*, *ntxxt2*, and *ntxxt1/2* seedlings. ** Statistically significant differences from K326 using Student’s *t*-test (*p* < 0.01), *** statistically significant differences from K326 using Student’s *t*-test (*p* < 0.001). **(F)** The growth rates indicated by plant height of K326, *ntxxt1*, *ntxxt2*, and *ntxxt1/2* mutants. These experiments were performed in triplicate. Error bars, mean ± SE.

The Arabidopsis *atxxt1/2* double mutant does not have a severe visible phenotype, even though no xyloglucan is synthesized (Cavalier et al., 2008; Xiao et al., 2016). Our *ntxxt1*, *ntxxt2*, and *ntxxt1/2* plants grew somewhat more slowly (Figures 3A–C,E,F) but only the *ntxxt2* and *ntxxt1/2* were appreciably smaller than wild-type at the adult stage (Figure 3C). Reduced growth, as indicated by leaf area, was more discernible in the *ntxxt1/2* double mutant than in either single mutant (Figure 3E). The growth rate indicated by plant height was also examined in the first 8 weeks (Figure 3F). The growth was reduced in *ntxxt1*, *ntxx2*, and *ntxxt1/2* mutants compared to K326. Moreover, the *ntxxt1/2* double mutant leaves were smaller and rounder than the leaves of K326 (Figure 3B). Similar visible differences in growth were also apparent in 4-week-old and adult plants (Figure 3B).

We also examined the root hair phenotype of the mutants. The root hairs of the *ntxxt1* and *ntxxt2* mutants are indistinguishable from those of K326 (Figure 3D). By contrast, the number of root hairs formed is substantially reduced in the double mutant and they are much shorter compared to K326 root hairs. The severe root hair phenotypes *ntxxt1/2* and *atxxt1/2* are similar to *atxxt1/2* with one exception: there is no bulging at the *ntxxt1/2* root hair base. Taken together these data show that eliminating both NtXXT1 and NtXXT2 has a discernible effect of the growth and development of tobacco plants.

### The Cell Walls of *ntxxt1/2* Lack Xyloglucan

**Figure 4:**
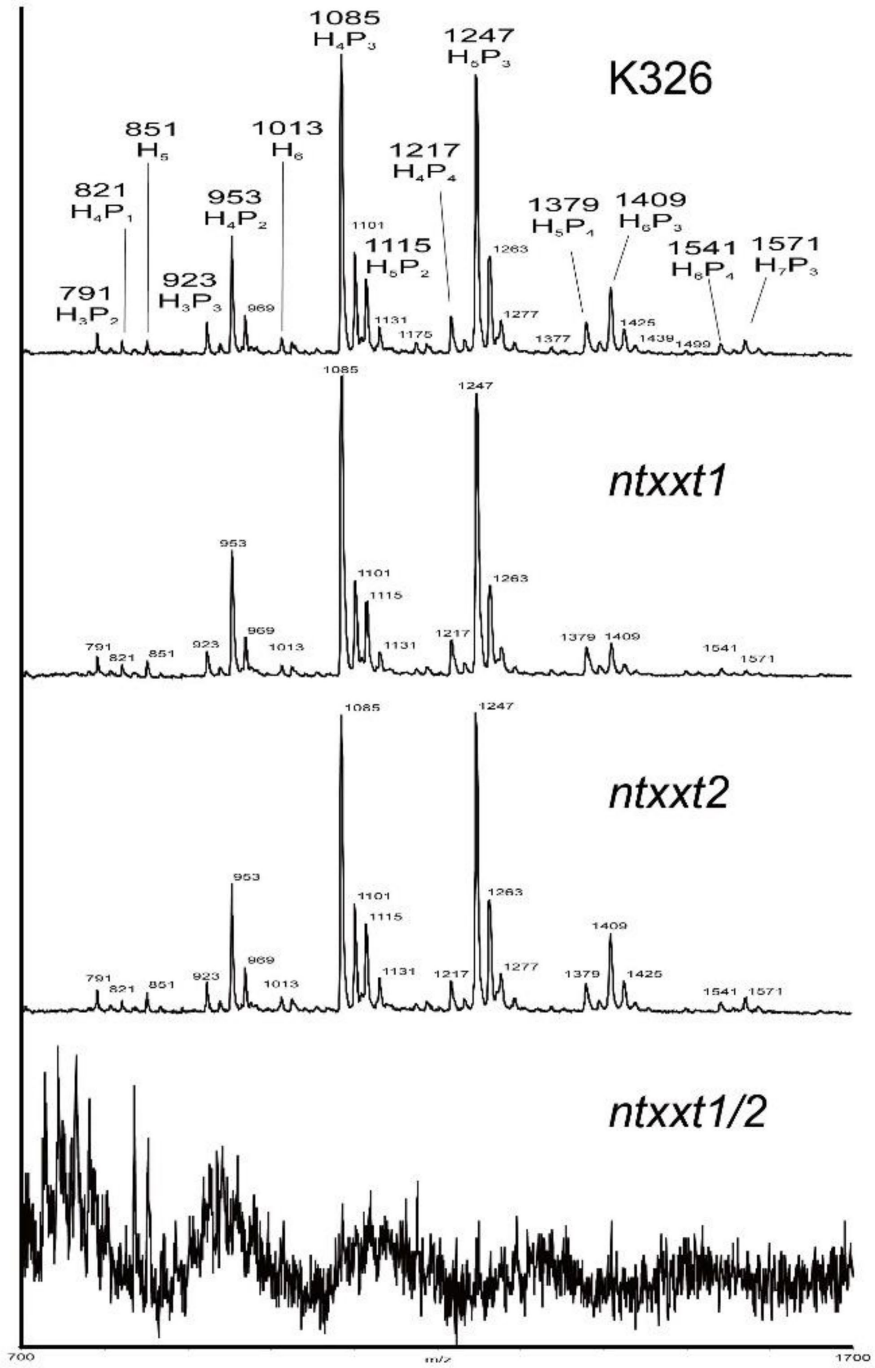
MALDI-TOF spectra of the XyG oligosaccharides generated from the 4 M KOH-soluble of AIR obtained from K326, *ntxxt1*, *ntxxt2*, and *ntxxt1/2* mutants plants.

**Figure 5:**
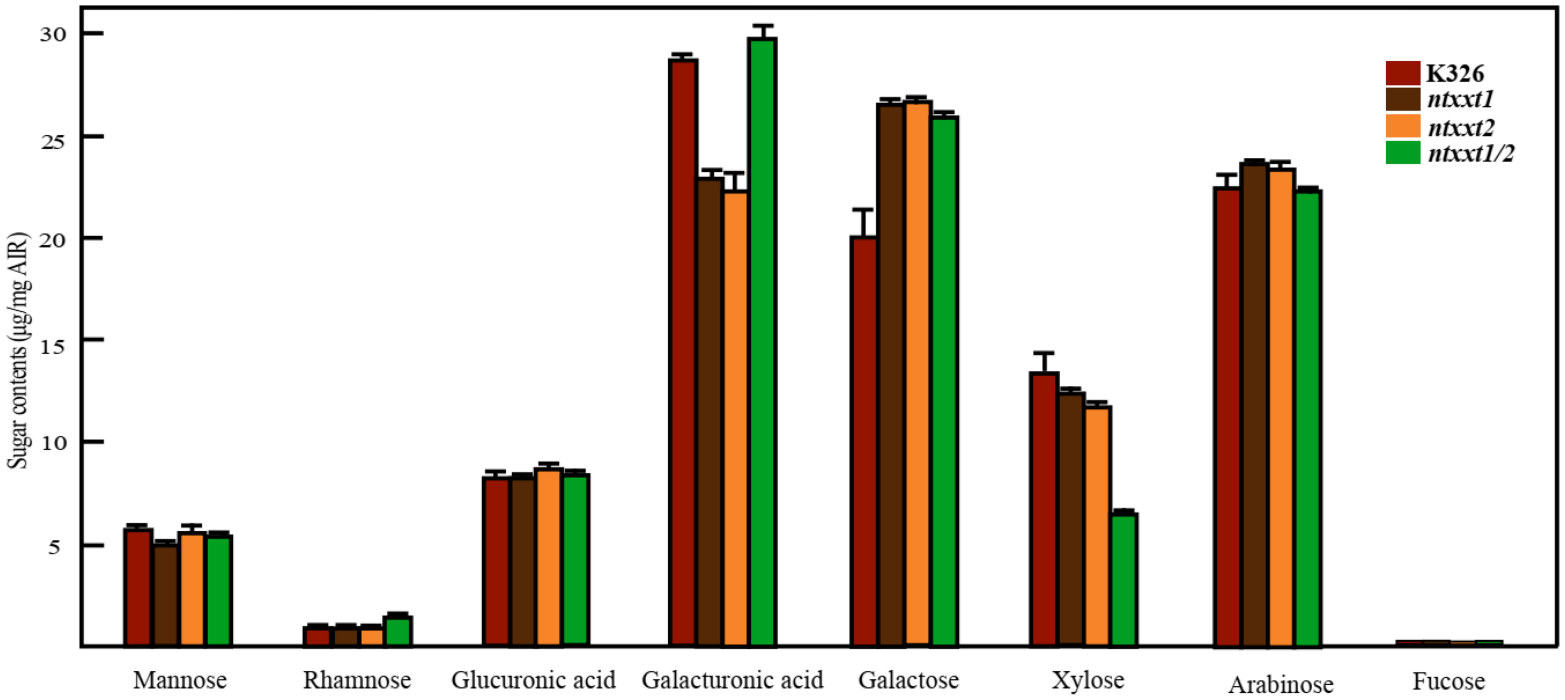
The glycosyl residue compositions of the AIR from K326, *ntxxt1*, *ntxxt2*, and *ntxxt1/2* leaves. These experiments were performed in triplicate. Error bars, mean ± SE.

The phenotypes of the *ntxxt1/2* mutant indicated that these two tobacco XXTs have a function similar to AtXXT1 and AtXXT2. To determine if xyloglucan is absent in *ntxxt1/2*, we treated the 4 M KOH-soluble material from the leaves of 4-week-old wild-type and mutant plants with XyG-specific endoglucanase (XEG) and analyzed the products formed with MALDI-TOF-MS (Kong et al., 2015). Our results show that there is no discernible difference in the relative abundance of XyG oligosaccharides in wild-type and *ntxxt1* or *ntxxt2* plants (Figure 4). However, no signals for XyG oligosaccharides were detected in *ntxxt1/2* mutant (Figure 4). We also found that the xylose content of the *ntxxt1/2* double mutant AIR was ~50% lower than that in the wild-type (Figure 5). The xylose content of the *ntxxt1* and *ntxxt2* single mutants decreased 6.7 and 11.2%, respectively (Figure 5). The xylose content of the AIR material was mainly from xyloglucan and xylan. Thus, the remaining 50% xylose content in *ntxxt1/2* double mutants is maybe from xylan. These results suggest that no detectable amounts of XyG is synthesized by the *ntxxt1/2* double mutant.

### Overexpression of *NtXXT1* or *NtXXT2* Rescues the Root Hair and Slow Growth Phenotype of the *atxxt1/2* Mutant

**Figure 6:**
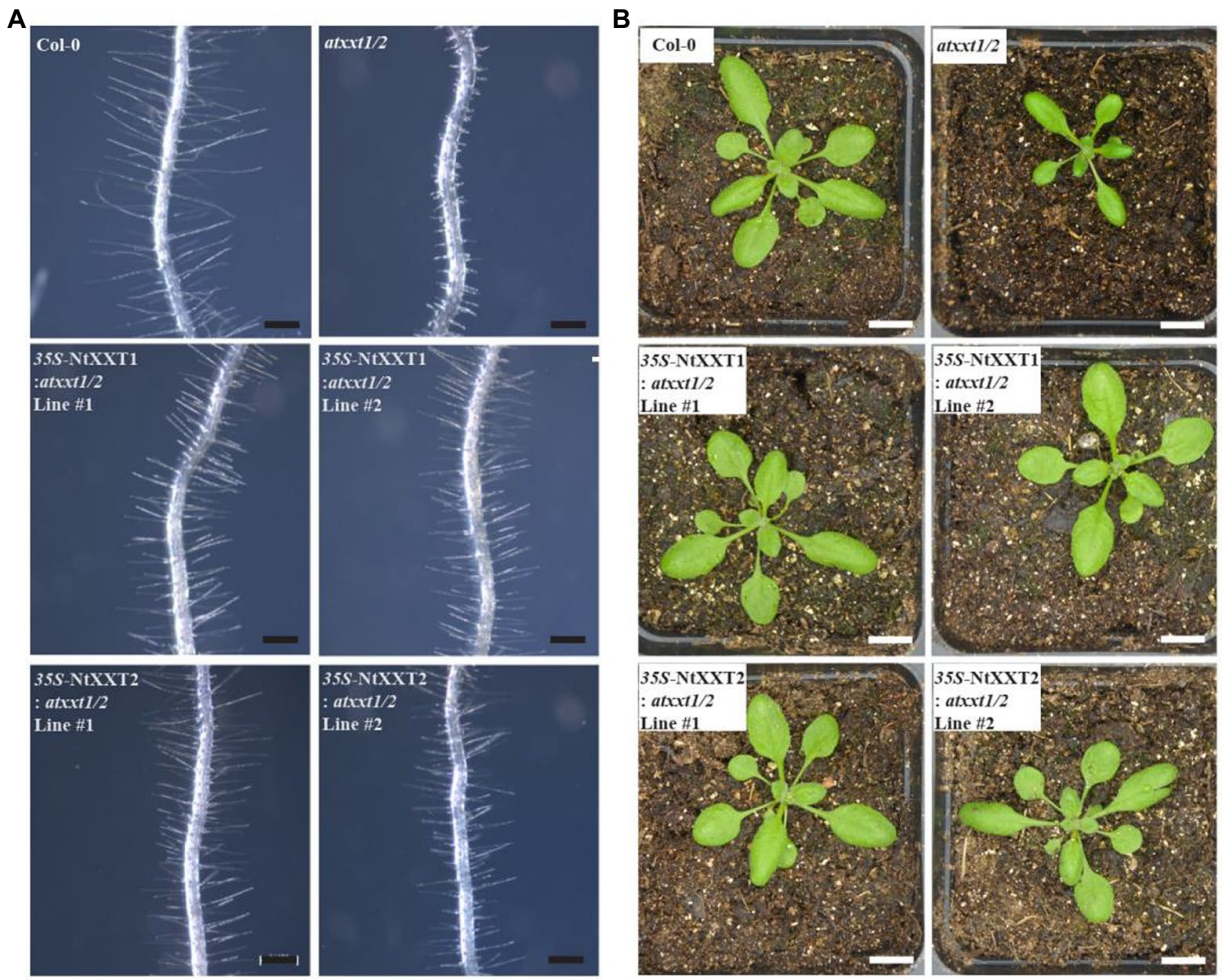
The root hair and plants phenotypes of complemented *atxxt1/2* lines. **(A)** The root hair phenotypes of 7 day-old Arabidopsis seedlings were observed. The root hairs of the *atxxt1/2* double mutant are far shorter than Col-0. Overexpressing *35S*-NtXXT1 or *35S*-NtXXT2 in *atxxt1/2* plants rescues the root hair defect of the *atxxt1/2* double mutant. Bar = 100 μm. **(B)** The 3-week-old plants phenotype was observed. The *atxxt1/2* double mutant plants were smaller than Col-0, and overexpressing *35S*-NtXXT1 or *35S*-NtXXT2 in *atxxt1/2* plants were recovered. Bar = 10 mm.

Our results have demonstrated that tobacco plants lacking both NtXXT1 and NtXXT2 have growth and root hair phenotypes and wall chemotypes that are similar to the Arabidopsis *xxt1/2* mutant plant. Thus, tobacco and Arabidopsis XXT1 and XXT2 likely have similar biochemical functions. To test this, we separately transformed the *atxxt1/2* double mutant with the coding sequences of NtXXT1 and NtXXT2 driven by a *35S* promoter. We obtained 10 complemented transgenic lines for *35S*-*NtXXT1* and 15 for *35S*-*NtXXT2*. The short root hair defect was rescued in all these transgenic lines (Figure 6A). And the slow growth phenotype of 3-week-old plants was also restored to WT (Figure 6B) in all these transgenic lines.

### XyG Deficiency in the Cell Wall Affects as Accumulation and Translocation

**Figure 7:**
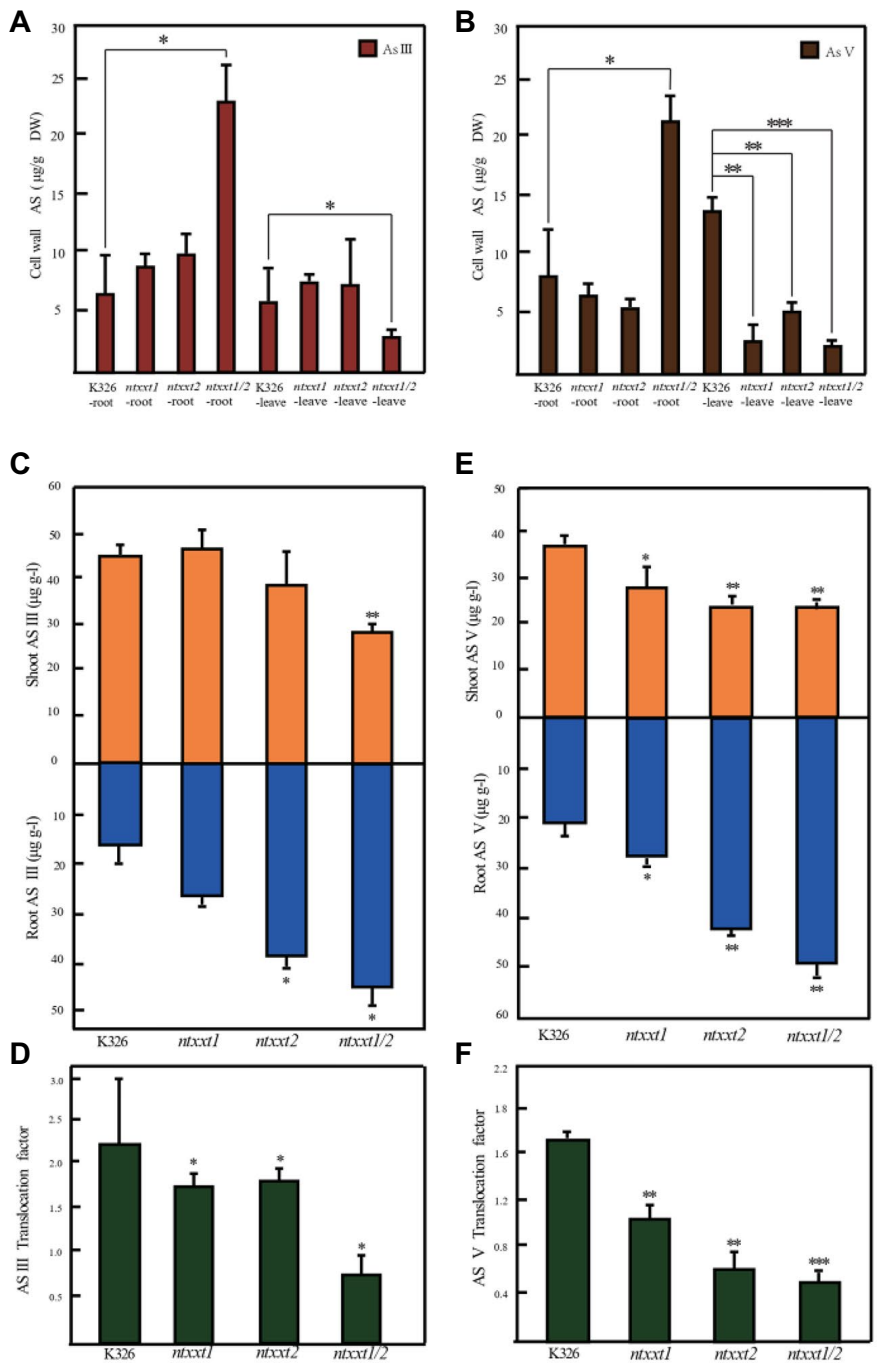
The As accumulation and translocation in *ntxxt1/2* double mutants grown with AsIII or AsV. **(A)** The amounts of As in the leaf and roots cell walls of K326, *ntxxt1*, *ntxxt2*, and *ntxxt1/2* grown with AsIII. **(B)** The amounts of As in the leaf and root cell walls of K326, *ntxxt1*, *ntxxt2*, and *ntxxt1/2* grown with AsV. **(C,E)** The amounts of As in shoots and roots of K326, *ntxxt1*, *ntxxt2*, and *ntxxt1/2* grown in the presence of AsIII or AsV. **(D,F)** Translocation factor (As concentration ratio in shoots to roots) for K326, *ntxxt1*, *ntxxt2*, and *ntxxt1/2* grown in the presence of AsIII or AsV. Experiments were performed in three biological replicates, and each biological replicates were performed in three technological replicates. Error bars, mean ± SE. * Statistically significant differences from K326 using Student’s *t*-test (*p* < 0.05), ** statistically significant differences from K326 using Student’s *t*-test (*p* < 0.01), *** statistically significant differences from K326 using Student’s *t*-test (*p* < 0.001).

It is not known if plants’ ability to accumulate As is altered by modifying its cell walls. Thus, we exposed wild-type and mutant tobacco plants to As to determine if the presence or absence of *NtXXT1* and *NtXXT2* affects As accumulation or translocation. A low As concentration (20 μM) was used as this does not cause severe growth inhibition in K326. We first determined the As concentration of root and leaf cell walls, since XyG is the predominant hemicellulose in tobacco primary cell walls and its absence may affect their mechanical and biochemical properties. The *ntxxt1/2* mutant grown with AsIII or AsV has increased amounts of As in its root cell walls and decreased amounts of As in its leaf cell walls compared to K326 (Figures 7A,B). The As contents of *ntxxt1* and *ntxxt2* root walls were not significantly different from K326, irrespective of whether they were grown with AsIII or AsV. Growing *ntxxt1/2* in the presence of AsIII and AsV also resulted in a significant reduction of leaf wall As. Leaf wall As was significantly lower than K326 for all three mutants grown in the presence of AsV.

The roots of *ntxxt1*, *ntxxt2*, and *ntxxt1/2* mutants grown with 20 μM AsIII, had 55, 118, and 154% more As, respectively, than K326 roots. Shoot As decreased by 7% in *ntxxt2* and by 49% in *ntxxt1/2* (Figure 7C). The calculated translocation factors (As concentration ratio in shoots to roots) for *ntxxt1*, *ntxxt2*, and *ntxxt1/2* were reduced by 21, 19, and 64%, respectively (Figure 7D). Similarly, As concentration increased in mutants grown with 20 μM AsV. Root As increased by 34, 107, and 140% in *ntxxt1*, *ntxxt2*, and *ntxxt1/2*, respectively. Shoot As decreased by 57, 53, and 75% in *ntxxt1*, *ntxxt2*, and *ntxxt1/2*, respectively (Figure 7E). The calculated translocation factors for *ntxxt1*, *ntxxt2*, and *ntxxt1/2* were reduced by 38, 62, and 71%, respectively (Figure 7F). Taken together these results suggest that As is retained more effectively in the roots of *ntxxt1/2* mutant and that As translocation from root to shoots is attenuated in the double mutants.

## Discussion

In this study, we used CRISPR-Cas9 technology to generate a tobacco mutant (*ntxxt1/2*) with cell walls that lack XyG. This modification resulted in root cell walls that accumulate more As than wild-type plants and thereby decreases the amounts of As that is translocated to the shoots (Figure 7). The relatively big increase of As in *ntxxt2* may be the result of the more decrease of xylose concentration. Thus, targeted engineering of cell walls provides an opportunity to generate plants capable of remediating soils contaminated with As.

Our data show that root cell walls lacking XyG bind more As than walls containing XyG. However, the nature of the interactions remains to be determined. In aqueous solutions, AsIII and AsV exist as protonated oxyanions with degrees of protonation that are pH-dependent (Ferguson and Gavis, 1972). AsIII has two pK_a_s (pH 9.2 and 12.1) whereas AsV has three (pH 2.2, 6.9, and 11.5). Thus, in the pH range of 4 to 8 typically encountered in the plant apoplast (Martiniere et al., 2013), AsIII will exist predominantly as the neutral arsenite H_3_AsO_3_, whereas AsV will exist as the negatively charged arsenates H_2_AsO_4_^−^ and HAsO_4_^2−^ (Wang and Mulligan, 2006). Arsenite has a high affinity for sulfhydryl groups (Shen et al., 2013), whereas arsenite and arsenate may form weak complexes with carboxylic acids, phenols, amines, and alcohols (Fakour and Lin, 2014).

There are only a limited number of studies that provide insight into the interactions between arsenites, arsenates, and cell wall polysaccharides. For example, As-pectin interactions have been proposed to occur *via* hydrogen-bonded bridging between the protons of the As species and the ionized carbonyl and carboxyl groups of the pectin (Fox et al., 2012). The As-pectin interaction has been reported to increase if the As exists as an iron oxide–arsenic complex (Wang and Mulligan, 2006; Fakour and Lin, 2014). Much more information on the interactions between As and polyelectrolytes has been obtained from studies of humic and fulvic acids (Wang and Mulligan, 2006; Fakour and Lin, 2014). These interactions may in part involve As anions forming metal bridges with the cations that are themselves bound to the polyelectrolyte. Thus, it is conceivable that As interacts with the metal cations bound to pectin. The galacturonic acid of cell wall pectin is typically associated with divalent cations, most notably calcium (Pérez et al., 2000; Braccini and Perez, 2001). Pectin has been reported to be a major contributor to As retention in rice cell walls *via* an interaction that is enhanced by silicates (Cui et al., 2020). Arsenic binding sites may also involve metal ions that form coordination complexes with the hydroxyl groups of mono- and polysaccharides (Angyal, 1989; Joly et al., 2020).

Xyloglucan is a quantitatively major polysaccharide in the primary cell wall of flowering plants. It has been proposed to interact with cellulose to form the major load-bearing network of these walls (Zhu et al., 2014). However, this notion has been challenged by the demonstration that an Arabidopsis mutant (*atxxt1/2*) unable to synthesize XyG does not have severe growth and developmental defects (Cavalier et al., 2008). Our study has shown that Tobacco plants lacking XyG also have no severe growth phenotypes. Thus, laboratory grown plants are able to survive in the absence of XyG. It is possible that in the absence of XyG, pectin takes on a greater share of the biomechanical load of the walls (Rui and Dinneny, 2020). Moreover, in XyG-deficient plants, the relative abundance or accessibility of wall pectin may increase and account for the increased amounts of bound As.

The absence of XyG is known to affect the organization of cellulose microfibrils and lead to a decrease in cellulose biosynthesis (Xiao et al., 2016). The cellulose microfibrils are largely parallel to one another, and the spacing between them is increased in the etiolated hypocotyls of the *atxxt1/2* mutant. Furthermore, the orientation of the cellulose microfibrils changes. In 3-day-old seedlings, the fibrils were predominantly transverse relative to the cell axis but longitudinal in 6-day-old *atxxt1/2* seedlings. Different orientations of cellulose may affect wall mechanics and properties. Thus, in the absence of XyG, cellulose organization may be altered and lead to changes in pectin organization within the wall. This pectin and other wall components may then become more accessible for binding with As.

We have provided evidence that no XyG is present in the walls of the *ntxxt1/2* double mutant. We expected that four tobacco orthologs of XXT1 and XXT2 would exist since *N. tabcum* is tetraploid. However, only two orthologs were identified and a tobacco mutant lacking functional *NtXXT1* and *NtXXT2* produced no discernible amounts of XyG. Thus, NtXXT1 and NtXXT2 must be the main xylosyltransferases involved in *N. tabcum* XyG biosynthesis. Both of these genes are expressed, albeit at different levels, in leaves, stems, veins and flowers. Nevertheless, *NtXXT1* expression is much higher than *NtXXT2* in these tissues (Figure 1B), which suggests that NtXXT1 is the predominant XyG-specific xylosyltransferase in most tissues. It is notable that reduced flowering and reduced plant height were more pronounced in the *ntxxt2* mutant than in *ntxxt1* (Figure 3C), which indicates that NtXXT2 may function predominantly in mature plants and at flowering time.

Elevated levels of As in the environment are harmful to virtually all organisms, limit agricultural productivity, and have severe effects on human health. Arsenic poisoning in humans often occurs *via* the food chain as plants grown in As-rich environments may accumulate toxic amounts of this element. Economically viable methods are required to remediate soils containing hazardous levels of As. Previous studies have shown that AsIII and AsV are transported into plant cells by aquaporin and phosphate transporters (Chen et al., 2017b, 2019, 2021). These transporters are required for normal plant growth and development. Thus, it is unlikely that they can be engineered to limit As uptake without affecting plant productivity. The cell wall is the first barrier to prevent As entry into plant cells. Our study and a previous report (Cavalier et al., 2008) have shown that this wall is malleable and that targeted elimination of XyG from the wall does not cause severe growth defects. We have shown that CRISPR-Cas9 technology can be used to generate tobacco plants that lack XyG in their cell walls. This modification allows the plant to accumulate more As in its root cell walls. Field studies are now required to demonstrate that such plants can be used to phytoremediate As contaminated soils.

## Data Availability Statement

The datasets presented in this study can be found in online repositories. The names of the repository/repositories and accession number(s) can be found in the article/supplementary material.

## Author Contributions

MW, XS, SG, PL, ZX, YK, and DY conceived the study. MW, XS, SG, PL, ZX, AD, HX, and RA performed the experiments. MW analyzed the data. XS prepared the figures. MW, YK, MO’N, and GZ drafted the manuscript. All authors discussed the results, edited the manuscript, and approved the final manuscript.

## Funding

This research was financially supported by the National Natural Science Foundation of China (32070330, 31670302, and 31470291), Yunnan Academy of Tobacco Agricultural Sciences (Nos. 2018530000241002 and 2019530000241003), Project of Shandong Natural Science Foundation (ZR2021QC138), the First-Class Grassland Science Discipline Program of Shandong Province, the Taishan Scholar Program of Shandong (to GZ), Grant DE-SC0008472 from the Division of Chemical Sciences, Geosciences, and Biosciences, Office of Basic Energy Sciences of the United States Department of Energy, and Foundation of Shandong Province Modern Agricultural Technology System (SDAIT-25-05).

## Conflict of Interest

The authors declare that the research was conducted in the absence of any commercial or financial relationships that could be construed as a potential conflict of interest.

## Publisher’s Note

All claims expressed in this article are solely those of the authors and do not necessarily represent those of their affiliated organizations, or those of the publisher, the editors and the reviewers. Any product that may be evaluated in this article, or claim that may be made by its manufacturer, is not guaranteed or endorsed by the publisher.

## Footnotes

^1^ http://www.arabidopsis.org

^2^ https://solgenomics.net/organism/Solanum_lycopersicum/genome

^3^ http://www.ebi.ac.uk/Tools/msa/clustalw2/

^4^ http://www.multicrispr.net/
